# Low early-pregnancy serum lipoprotein(a) as an independent associated factor for gestational diabetes mellitus: a retrospective cohort study

**DOI:** 10.3389/fendo.2026.1780579

**Published:** 2026-04-13

**Authors:** Feifei Wang, Jiangnan Wu, Hongling Li, Shijie Gao, Zhuohan Zheng, Zhiheng Wang, Jing Gao

**Affiliations:** 1 Department of Laboratory Medicine, Obstetrics & Gynecology Hospital of Fudan University, Shanghai Key Lab of Reproduction and Development, Shanghai Key Lab of Female Reproductive Endocrine Related Diseases, Shanghai, China; 2 Department of Laboratory Medicine, Obstetrics and Gynecology Hospital of Fudan University, Yangtze River Delta Integration Demonstration Zone (QingPu), Shanghai, China; 3 Clinical Research Unit, Obstetrics & Gynecology Hospital of Fudan University, Shanghai Key Lab of Reproduction and Development, Shanghai Key Lab of Female Reproductive Endocrine Related Diseases, Shanghai, China; 4 Radiotherapy Department, Obstetrics and Gynecology Hospital of Fudan University, Yangtze River Delta Integration Demonstration Zone (QingPu), Shanghai, China

**Keywords:** early-pregnancy, gestational diabetes mellitus, lipoprotein(a), propensity score matching, retrospective cohort study

## Abstract

**Background:**

Dyslipidemia plays a significant role in the pathogenesis of gestational diabetes mellitus (GDM). However, the association between lipoprotein(a) (Lp(a)), a known predictor of cardiovascular disease, and GDM remains unclear. This study aimed to investigate the association between serum Lp(a) levels within the first 20 weeks of gestation and the subsequent risk of developing GDM.

**Methods:**

We conducted a retrospective cohort study of 14,527 pregnant women between September 2021 and January 2024. Using propensity score matching (PSM), 922 women with GDM were matched with 922 women with normal glucose tolerance (NGT). Lp(a) levels measured within the first 20 gestational weeks were compared between the two groups. A logistic regression model was employed to assess the association between Lp(a) levels and the later development of GDM. Additionally, restricted cubic spline regression models were applied to examine whether a nonlinear relationship existed between Lp(a) and GDM risk. Finally, sensitivity analyses across subgroups were conducted to assess the robustness of the findings.

**Results:**

Serum Lp(a) levels within the first 20 weeks of gestation were significantly lower in the GDM group compared to the NGT group (*p* = 0.013). After adjustment for potential confounders, compared to the group with Lp(a) ≥300 mg/L, those with Lp(a) levels of 50–300 mg/L and ≤50 mg/L exhibited a higher risk of developing GDM, with adjusted odds ratios of 1.354 (95% CI: 1.059-1.732; *p* = 0.016) and 1.454 (95% CI: 1.073-1.970; *p* = 0.016), respectively. Subgroup analysis indicated that maternal age, pre-pregnancy body mass index (BMI) and gestational weeks of Lp(a) measurement did not significantly influence the association between Lp(a) levels and GDM.

**Conclusion:**

Low Lp(a) levels within the first 20 weeks of gestation were associated with the subsequent development of GDM, independent of maternal age and pre-pregnancy BMI.

## 1 Introduction

Gestational diabetes mellitus, the most prevalent metabolic disorder during pregnancy, is typically diagnosed in the mid-to-late gestational period (1) and affects approximately 15% of pregnant women worldwide (2). GDM results from an inadequate compensatory insulin secretion in the context of pregnancy-induced physiological insulin resistance (3, 4). This condition is clinically significant as it elevates the risk of serious maternal complications, such as cesarean delivery and shoulder dystocia, and predisposes the offspring to macrosomia and neonatal hypoglycemia (5). Moreover, women with a history of GDM face a substantially increased susceptibility to developing type 2 diabetes mellitus (T2DM) and cardiovascular disease (CVD) later in life (6), while their offspring are at a heightened risk of early-onset obesity and T2DM (7). The standard for GDM diagnosis, as recommended by the International Association of Diabetes and Pregnancy Study Groups, is a 75-g 2-hour oral glucose tolerance test (OGTT) performed during the mid-trimester (24–28 weeks of gestation) (8).

Established risk factors for GDM include a family history of diabetes (9), elevated BMI (10, 11), Western-style diet (12), specific ethnic backgrounds (13), advanced maternal age (14), and multiparity (15). Furthermore, dyslipidemia in GDM patients has been extensively documented. This aberrant lipid profile is characterized by alterations in triglycerides (TG), total cholesterol (TC), low-density lipoprotein cholesterol (LDL-C), very-low-density lipoprotein cholesterol (VLDL-C), high-density lipoprotein cholesterol (HDL-C), and free fatty acids (FFA) (16–19), all of which play a significant role in the pathogenesis of GDM (20, 21).

Structurally, Lp(a) is a complex particle formed by the disulfide linkage of apolipoprotein A to apolipoprotein B-100 on an LDL core (22). Lp(a) concentrations exhibit remarkable inter-individual variability, spanning over a 1000-fold range from below 0.1 mg/dL to over 300 mg/dL. Importantly, serum Lp(a) levels remain relatively stable within an individual and are largely unaffected by age, sex, smoking, diet, environment, or lipid metabolism, being primarily genetically determined (23). It is well-established that elevated plasma Lp(a) is causally associated with various CVD, including myocardial infarction, ischemic stroke, calcific aortic valve disease, and chronic heart failure, rendering it a strong independent risk factor for CVD (24). During normal pregnancy, the maternal metabolic system undergoes adaptive changes, with carbohydrate and lipid metabolism being tightly coordinated. As a key component of lipid metabolism, Lp(a) may theoretically disrupt glucose metabolic homeostasis and contribute to the pathogenesis of GDM. However, the association between Lp(a) during pregnancy and GDM remains inconclusive.

Therefore, this study aimed to address this knowledge gap by comparing serum Lp(a) levels prior to 20 weeks of gestation between women with and without subsequent GDM. Utilizing PSM to control for potential confounders, we investigated the association between early-pregnancy Lp(a) and the subsequent risk of GDM. The findings are expected to provide a scientific basis for optimizing prenatal care and improving maternal and neonatal outcomes.

## 2 Methods

### 2.1 Design and participants

We conducted a retrospective cohort study involving 14,527 women with singleton pregnancies who initiated prenatal care at the Obstetrics and Gynecology Hospital of Fudan University (Shanghai, China) between September 2021 and January 2024. Participants were enrolled at the time of their first prenatal registration at our institution. The exclusion criteria were as follows: pre-pregnancy diabetes, GDM before 24 gestational weeks, fasting plasma glucose ≥5.1 mmol/L detected at the initial prenatal visit, Lp(a) measurement beyond 20 gestational weeks, malignancy, severe metabolic diseases (including Cushing’s syndrome, hyperthyroidism, and hypothyroidism), failure to provide written informed consent, and absence of complete maternal and neonatal records. Maternal and neonatal clinical data and laboratory results were retrieved from the Hospital Information System and Laboratory Information System, respectively. This study was conducted in accordance with the principles of the Declaration of Helsinki and was approved by the Institutional Review Board of the Obstetrics and Gynecology Hospital of Fudan University (Approval No.: 2020-49).

### 2.2 Definition of GDM

The primary outcome of this study was the diagnosis of GDM, which was defined according to the criteria established by the International Association of Diabetes and Pregnancy Study Groups (25). During 24 to 28 weeks of gestation, all participants underwent a 75-g OGTT. GDM was diagnosed if any of the following plasma glucose values were met or exceeded (1): fasting plasma glucose ≥5.1 mmol/L (2); 1-hour plasma glucose ≥10.0 mmol/L; or (3) 2-hour plasma glucose ≥8.5 mmol/L. Otherwise, participants were classified as having NGT.

### 2.3 Lp(a) measurement and grouping

The primary exposure variable was the first available serum Lp(a) measurement obtained before 20 weeks of gestation. In our hospital, Lp(a) was measured as part of routine early prenatal screening for all women. Serum Lp(a) concentrations were measured using a latex-enhanced immunoturbidimetric assay on a Hitachi 7180 automatic biochemical analyzer (WAKO). Lp(a) levels were tested using commercial assay kits (Ranos Lp(a) test, Medconn biotechnology Co. Ltd, Shanghai, China), which was calibrated with a dedicated kit (Lp(a) Calibrator, Registration No.: 20212400234). Results are reported in mg/L. Critically, this assay utilizes antibodies that are insensitive to apolipoprotein(a) isoform sizes, thereby ensuring accurate and comparable quantification across different Lp(a) phenotypes. The inter- and intra-assay coefficients of variation were both <5%. The reference range for Lp(a) in a healthy population is 0–300 mg/L, and levels below 5 mg/dL have been associated with a significantly increased risk of type 2 diabetes (26). Based on their serum Lp(a) levels, participants were categorized into three groups: ≤50 mg/L, 50–300 mg/L, and ≥300 mg/L.

### 2.4 Covariates

The following covariates were collected in this study: maternal age, parity, pre-pregnancy BMI, gestational age at blood sampling, family history of metabolic diseases, alanine aminotransferase (ALT), uric acid (UA), and creatinine (Cr).

### 2.5 Data analysis

**Figure 1:**
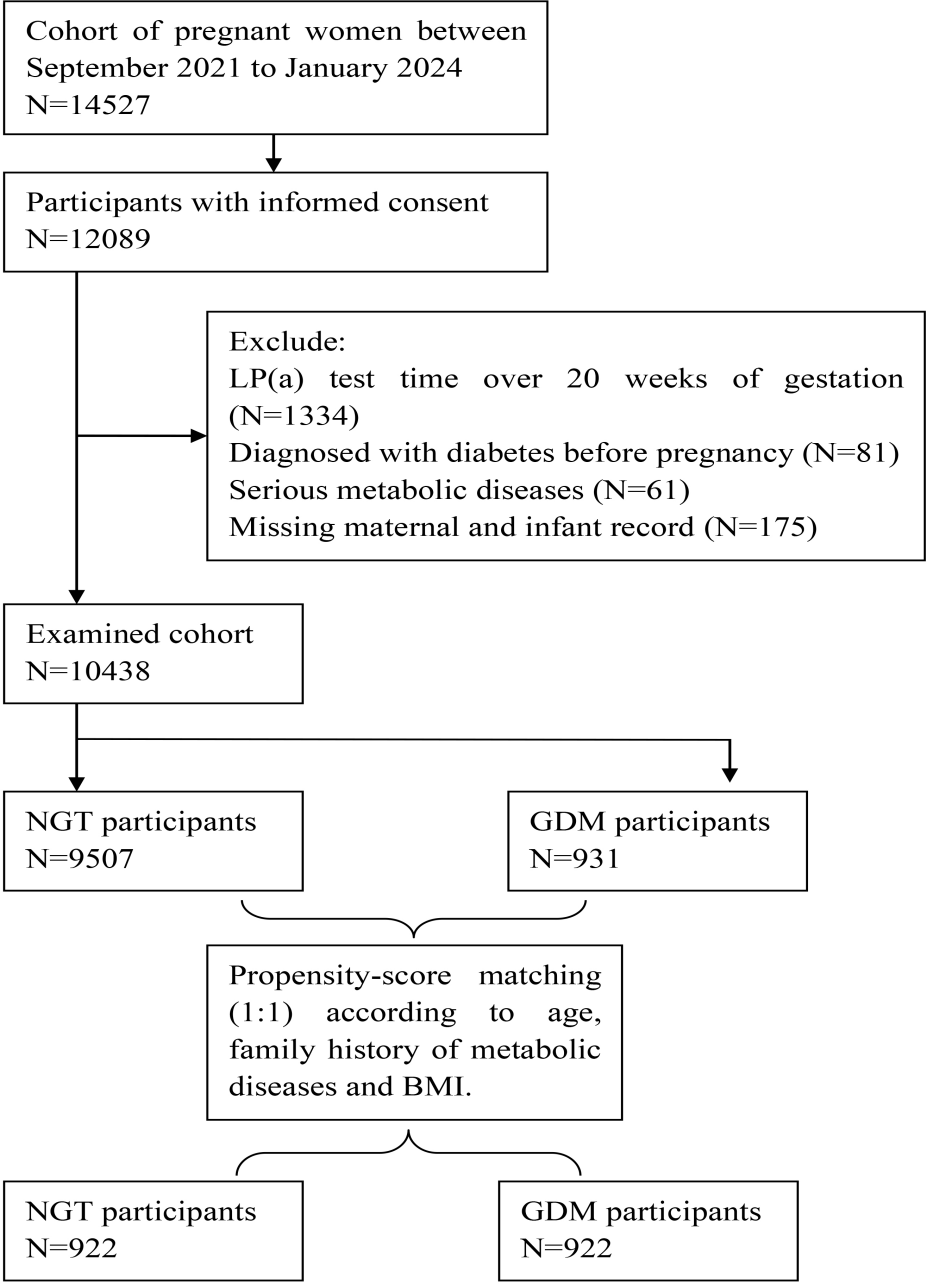
The flowchart of the selection of GDM participants and the matched control participants.

We employed PSM to identify a cohort of 922 women with GDM and 922 women with NGT for our final analysis. To adjust for baseline differences, the following potential confounders associated with GDM were selected as matching variables: maternal age, pre-pregnancy BMI, and gestational weeks at Lp(a) measurement. GDM and NGT participants were matched in a 1:1 ratio without replacement using the nearest-neighbor algorithm, with a caliper width of 0.01 of the logit of the propensity score. A flowchart illustrating the selection of the GDM cohort and the matched control group for analysis is presented in **Figure 1**.

Normally distributed data are presented as mean ± standard deviation, while non-normally distributed continuous variables are expressed as median (interquartile range). Differences between groups were assessed using independent samples t-tests for normally distributed continuous variables, Mann-Whitney U tests for non-normally distributed continuous variables, and Chi-square or Fisher’s exact tests for categorical variables, as appropriate. The association between Lp(a) and GDM was evaluated using binary logistic regression analysis, which estimated odds ratios (OR) before and after adjustment for potential confounding factors. Restricted cubic spline (RCS) regression models were run to explore nonlinear association between continuous Lp(a) levels and risk of GDM. In the RCS analysis, the log-transformed values of Lp(a) were categorized into equal-width intervals. The 10th, 50th, 90th, and 95th percentiles (P10: 37 mg/L, P50: 120 mg/L, P90: 481 mg/L, P95: 658 mg/L) were selected as the knot locations. The interval with the highest frequency (79.44–100 mg/L) was used as the reference group.

To verify the robustness of our findings, we conducted sensitivity analyses across subgroups stratified by gestational weeks of Lp(a) measurement (<12 weeks, 12–20 weeks), maternal age (<35 years, ≥35 years), and pre-pregnancy BMI (<23 kg/m², ≥23 kg/m²). The 12-week cutoff was selected because it represents the established demarcation between the first and second trimesters in clinical practice, during which substantial changes occur in maternal metabolic and hormonal environments (27). Interaction terms were tested to evaluate potential heterogeneity in the Lp(a)-GDM association across these subgroups. It should be noted that these subgroup analyses were exploratory in nature, and the statistical power may have been insufficient to detect true interaction effects. Therefore, the findings should be considered hypothesis-generating.

A two-sided *p*-value of < 0.05 was considered statistically significant for all analyses. All statistical computations were performed using IBM SPSS Statistics (Version 25.0; IBM, NY, US). Propensity score matching was conducted with the PSMATCH 3.04 extension (R-language based) within SPSS. Subsequent statistical analyses were performed on the matched dataset.

## 3 Results

### 3.1 Baseline characteristics

Of the initial 14,527 pregnant women assessed, 10,438 met the inclusion and exclusion criteria, comprising 931 women with GDM and 9,507 with NGT. Subsequently, propensity score matching (1:1) was performed based on pre-pregnancy BMI, maternal age, and gestational weeks of Lp(a) measurement, yielding a final matched cohort of 922 GDM patients and 922 NGT controls for this study (**Figure 1**).

A comparison of all variables between the entire GDM and NGT cohorts is summarized in **Table 1**. As anticipated, after PSM, no significant differences were observed in age or BMI between the GDM and NGT groups (*p* > 0.05), indicating that the influence of these confounders was sufficiently reduced. In the PSM cohort, significant differences between the GDM and NGT groups were found for gestational weeks of Lp(a) measurement, parity, fasting blood glucose, TG, HDL, LDL, apolipoprotein A (APO-A), apolipoprotein B (APO-B), Lp(a), and UA (*p* < 0.05). In contrast, no significant differences were detected for ALT, TC, Cr (*p* > 0.05). Notably, serum Lp(a) levels were significantly lower in the GDM group [100 (57–218) mg/L] than in the NGT group [116.5 (60–270) mg/L; *p* = 0.013]. Furthermore, the GDM group exhibited significantly higher serum levels of TG, LDL, APO-B, and UA, but lower levels of HDL and APO-A compared to the NGT group (all *p*-values < 0.05).

**Table 1:** Baseline characteristics before and after PSM.

Characteristic	Cohort before matching		Cohort after matching	
	NGT N=9507	GDM N=931	NGT N=922	GDM N=922
Age (years)	30.82 ± 3.87	32.25 ± 3.97**	32.06 ± 3.77	32.21 ± 3.95
Pre-pregnancy BMI (kg/m^2^)	20.70(19.14-22.58)	21.97(20.08-23.94)**	22.29 ± 3.11	22.28 ± 3.05
Family history of metabolic diseases				
No	6539(68.78%)	568(61.01%)**	546(59.2%)	566(61.4%)
Yes	2968(31.22%)	363(38.99%)**	376(40.8%)	356(38.6%)
Gestational weeks of Lp(a) measurement	10.59 ± 2.01	10.58 ± 1.96	10.34 ± 2.01	10.58 ± 1.95**
Parity				
Primipara	7563(79.6%)	712(76.5%)*	770(83.5%)	705(76.5%)**
Multipara	1944(20.4%)	219(23.5%)*	152(16.5%)	217(23.5%)**
Fasting blood glucose (mmol/L)	4.46 ± 0.31	4.57 ± 0.36**	4.48 ± 0.31	4.57 ± 0.36**
ALT (U/L)	13 (10-19)	15 (11-22)**	14 (11-20)	15(11-21)
TC (mmol/L)	4.50 ± 0.74	4.64 ± 0.74**	4.65 ± 0.79	4.64 ± 0.74
TG (mmol/L)	1.25 ± 0.55	1.49 ± 0.80**	1.40 ± 0.81	1.49 ± 0.80*
HDL (mmol/L)	1.45 ± 0.44	1.48 ± 0.42*	1.65 ± 0.40	1.49 ± 0.42**
LDL (mmol/L)	2.67 ± 0.60	2.77 ± 0.61**	2.64 ± 0.73	2.77 ± 0.61**
APO-A (g/L)	1.56 ± 0.35	1.62 ± 0.35**	1.73 ± 0.31	1.62 ± 0.35**
APO-B (g/L)	0.77 ± 0.16	0.82 ± 0.17**	0.79 ± 0.19	0.82 ± 0.17**
Lp(a) (mg/L)	121 (64-259)	100 (57-217)**	116.5(60-270)	100(57-218)*
Cr (μmol/L)	43.90 ± 5.72	43.84 ± 5.88	43.97 ± 5.93	43.79 ± 5.85
UA (μmol/L)	209.4 ± 43.6	226.2 ± 49.3**	220.33 ± 47.38	225.75 ± 49.18*

Data are presented as the mean ± standard deviation, median (interquartile range) or number (Frequency). Data on age, gestational weeks of Lp(a) measurement, fasting blood glucose, TC, TG, HDL, LDL, APO-A, APO-B, Cr and UA were compared using the t-test; Pre-pregnancy BMI, ALT and Lp(a) were compared using the Mann-Whitney U test; family history of metabolic diseases and parity were compared using the chi-square test and Fisher’s exact test. *p < 0.05 and **p < 0.01 compared with the control groups. The standardized mean differences for age, pre-pregnancy BMI, and gestational weeks at Lp(a) measurement before and after PSM were 0.039, 0.003, and 0.121, respectively.

### 3.2 The association between Lp(a) and the risk of GDM

Subsequently, the study participants were stratified based on serum Lp(a) levels into three categories: ≤50 mg/L, 50 to 300 mg/L, and ≥300 mg/L. The association between Lp(a) and GDM was analyzed using logistic regression. Compared to the group with Lp(a) ≥300 mg/L, a significant inverse trend was observed for GDM risk with decreasing Lp(a) levels (*p* for trend = 0.045). Specifically, women with Lp(a) levels of 50–300 mg/L exhibited a higher risk of GDM (OR 1.290, 95% CI 1.015–1.641; *p* = 0.037), while those with Lp(a) ≤50 mg/L demonstrated the highest risk (OR 1.352, 95% CI 1.010–1.811; *p* = 0.043). After adjusting for potential confounders, the results remained consistent. The adjusted odds ratios for GDM were 1.354 (95% CI 1.059-1.732; *p* = 0.016) in the 50–300 mg/L group and 1.454 (95% CI 1.073-1.970; *p* = 0.016) in the ≤50 mg/L group, compared to the reference group (Lp(a) ≥300 mg/L). These findings indicate that low serum Lp(a) levels in early pregnancy were independently associated with GDM (**Table 2**).

**Table 2:** Associations of serum Lp(a) concentrations (mg/L) with the risk of gestational diabetes mellitus.

Lp (a) (mg/L)	NGT (N = 922)	GDM (N = 922)	Odds ratio	*p*	Adjusted^a^ odds ratio	*p*
≥300	196 (21.3%)	158 (17.1%)	Reference		Reference	
50-300	548 (59.4%)	570 (61.8%)	1.290 (1.015-1.641)	0.037	1.354 (1.059-1.732)	0.016
<50	178 (19.3%)	194 (21.1%)	1.352 (1.010-1.811)	0.043	1.454 (1.073-1.970)	0.016
*p*_trend_				0.045		0.000

Values are given as odds ratio (95% CI (confidence interval)). ^a^Adjusted for age, pre-pregnancy BMI, family history of metabolic diseases, gestational weeks of Lp(a) measurement, parity, ALT, Cr, UA.

**Figure 2:**
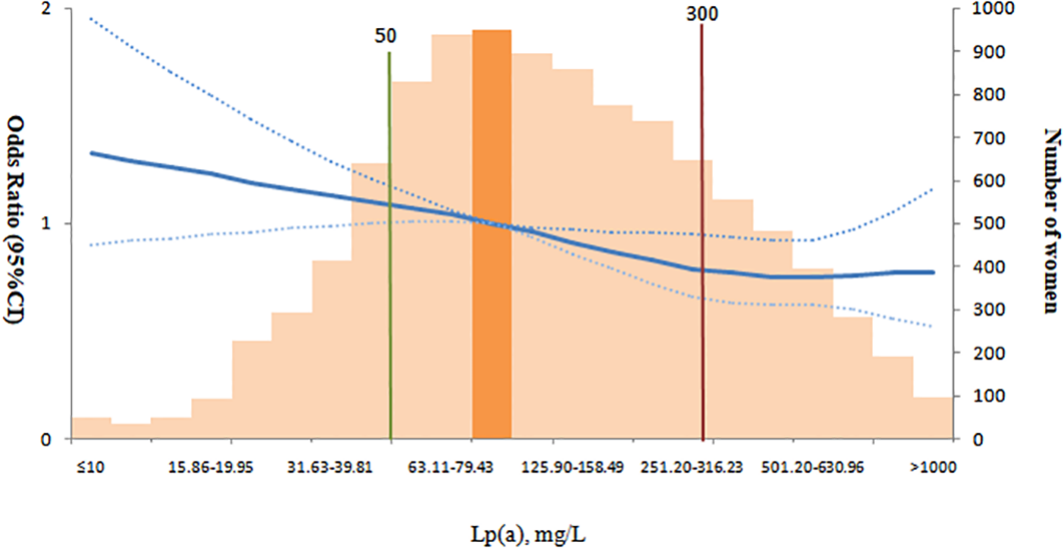
In the RCS analysis, the log-transformed values of Lp(a) were categorized into equal-width intervals. The 10th, 50th, 90th, and 95th percentiles (P10: 37 mg/L, P50: 120 mg/L, P90: 481 mg/L, P95: 658 mg/L) were selected as the knot locations. The interval with the highest frequency (79.44–100 mg/L) was used as the reference group. Sample sizes at the extremes of the RCS curve were small (e.g., <100 pregnant women for Lp(a) <30 mg/L or >1000 mg/L), leading to wide 95% CIs; caution is needed when interpreting results in these ranges.

Furthermore, restricted cubic spline analysis revealed a nonlinear relationship between Lp(a) levels and GDM risk (*p* for nonlinearity = 0.01). The highest risk of GDM was observed at lower Lp(a) concentrations, with a gradual decline in risk as Lp(a) levels increased. The association plateaued once Lp(a) levels exceeded approximately 300 mg/L, beyond which further increases in Lp(a) were not associated with additional changes in GDM risk (**Figure 2**).

### 3.3 Sensitivity analyses by subgroups

Subsequently, sensitivity analyses were performed in subgroups stratified by age, BMI, and timing of Lp(a) measurement, which revealed no interaction effects of these factors on the association between Lp(a) and GDM (all *p* for interaction > 0.05). After adjustment for potential confounders, a significant association between Lp(a) levels and GDM was observed in the subgroup with Lp(a) measured before 12 weeks of gestation and the subgroup aged < 35 years. Specifically, in the adjusted model for the early gestation subgroup (measurement prior to 12 weeks), the GDM risk was 1.392-fold higher (95% CI 1.046-1.852; *p* = 0.023) among women with Lp(a) levels of 50–300 mg/L and 1.560-fold higher (95% CI 1.103-2.206; *p* = 0.012) among those with Lp(a) ≤50 mg/L, compared to the reference group (Lp(a) ≥300 mg/L). In contrast, no significant association was observed in subgroups with Lp(a) measured after 12 weeks of gestation, BMI ≥ 23 kg/m², or age ≥ 35 years (**Table 3**).

**Table 3:** Subgroup analysis of GDM and Lp(a).

Characteristic	NGT (N = 922)	GDM (N = 922)	Crude OR	*p*	Adjusted^a^ OR	*p*
Gestational weeks at blood sampling						
≤12 weeks						
LP(a)≥300 mg/L	154(16.7%)	114(12.4%)	Reference		Reference	
50≤LP(a)<300 mg/L	437(47.4%)	429(46.5%)	1.326(1.006-1.748)	0.045	1.392(1.046-1.852)	0.023
LP(a)<50 mg/L	148(16.1%)	158(17.1%)	1.442(1.037-2.006)	0.030	1.560(1.103-2.206)	0.012
>12 weeks						
LP(a)≥300 mg/L	42(4.6%)	44(4.8%)	Reference		Reference	
50≤LP(a)<300 mg/L	111(12.0%)	141(15.3%)	1.213(0.742-1.980)	0.441	1.112(0.670-1.844)	0.681
LP(a)<50 mg/L	30(3.3%)	36(3.9%)	1.145(0.602-2.179)	0.679	1.037(0.529-2.031)	0.916
BMI						
<23 kg/m²						
LP(a)≥300 mg/L	121(13.1%)	103(11.2%)	Reference		Reference	
50≤LP(a)<300 mg/L	354(38.4%)	378(41.0%)	1.254(0.929-1.693)	0.139	1.318(0.970-1.793)	0.078
LP(a)<50 mg/L	113(12.3%)	119(12.9%)	1.237(0.856-1.787)	0.257	1.396(0.950-2.050)	0.089
≥23 kg/m²						
LP(a)≥300 mg/L	75(8.1%)	55(6.0%)	Reference		Reference	
50≤LP(a)<300 mg/L	194(21.0%)	192(20.8%)	1.350(0.904-2.016)	0.143	1.357(0.896-2.054)	0.149
LP(a)<50 mg/L	65(7.0%)	75(8.1%)	1.573(0.973-2.545)	0.065	1.489(0.900-2.461)	0.121
Age						
≤35 years						
LP(a)≥300 mg/L	160(17.4%)	131(14.2%)	Reference		Reference	
50≤LP(a)<300 mg/L	449(48.7%)	449(48.7%)	1.221(0.937-1.593)	0.140	1.318(1.005-1.730)	0.046
LP(a)<50 mg/L	137(14.9%)	151(16.4%)	1.346(0.971-1.867)	0.075	1.505(1.072-2.114)	0.018
>35 years						
LP(a)≥300 mg/L	36(3.9%)	27(2.9%)	Reference		Reference	
50≤LP(a)<300 mg/L	99(10.7%)	121(13.1%)	1.630(0.926-2.868)	0.090	1.464(0.813-2.635)	0.204
LP(a)<50 mg/L	41(4.4%)	43(4.7%)	1.398(0.725-2.698)	0.317	1.268(0.632-2.545)	0.503

a Adjusted for age, pre-pregnancy BMI, family history of metabolic diseases, gestational weeks of Lp(a) measurement, parity, ALT, Cr, UA.

## 4 Discussion

### 4.1 Main findings

This study represents the first investigation to evaluate the association between first-trimester Lp(a) levels and GDM using a retrospective cohort study with PSM design. In this PSM study, we found that serum Lp(a) levels before 20 weeks of gestation were significantly lower in the GDM group compared to the NGT group. Lower serum Lp(a) levels were associated with an increased risk of GDM in the mid-to-late stages of pregnancy. Individuals with Lp(a) levels less than 50 mg/L exhibited the highest risk of developing GDM (OR 1.454, 95% CI 1.073-1.970; *p* = 0.016).

### 4.2 Comparison with previous studies

While there is growing interest in lipid profile changes during pregnancy in women with GDM, findings from existing literature remain contentious. A systematic review (28) reported that, compared to non-GDM women, those with GDM exhibited significantly elevated TG levels throughout gestation and significantly reduced HDL-C levels in the mid-to-late trimesters, with no differences in TC or LDL-C levels. In contrast, a meta-analysis (29) concluded that the GDM group had higher TG levels, elevated LDL-C in the first trimester, and increased TC along with decreased HDL in the early to mid-trimester. Our conclusions are derived from measurements taken within the first 20 weeks of gestation, encompassing both the first and early second trimesters. As presented in **Table 1**, compared to the NGT group, women who developed GDM showed significantly higher serum levels of TG, Apo-B, and LDL, alongside lower levels of HDL and Apo-A during early pregnancy, with no difference in TC. These results reinforce that alterations in maternal lipid metabolism precede the clinical onset of GDM.

Current evidence regarding the association between Lp(a) levels and GDM remains limited. A previous machine learning study (30) identified first-trimester Lp(a) as among the most promising predictors for GDM, with a predictive value even surpassing that of other apolipoproteins. In contrast, a case-control study involving 64 GDM patients and 165 controls with NGT (31) reported no significant difference in serum Lp(a) concentrations between the groups at 24–28 weeks of gestation. Conversely, a prospective cohort of 445 pregnant women (32) suggested that elevated Lp(a) in the first trimester is a risk factor for GDM. Notably, our study, derived from a large cohort of 10,438 individuals, is the first to employ a retrospective cohort study with PSM design, including 922 GDM patients and 922 NGT controls. Our sample size is substantially larger than those of previous studies, thereby providing more robust evidence. We conclude that low serum Lp(a) levels before 20 weeks of gestation were associated with higher odds of GDM. Notably, our results demonstrate that individuals with Lp(a) levels ≤50 mg/L face the highest risk of developing GDM, with a strikingly 1.454-fold higher risk compared to those with Lp(a) levels ≥300 mg/L. This inverse relationship is consistent with existing literature on T2DM. Previous studies (33, 34) have established an inverse association between Lp(a) and T2DM risk, where concentrations below 4 mg/dL (33) or 7 mg/dL (34) were significantly associated with increased incidence and highest risk of T2DM, respectively. To our knowledge, this is the first study to identify Lp(a) ≤50 mg/L as the threshold conferring the greatest association with GDM. Based on this finding, we propose that Lp(a) holds promise as a potential target for GDM prevention. Whether targeted interventions in pregnant women with Lp(a) levels ≤50 mg/dL prior to the onset of GDM can effectively reduce the disease incidence warrants further investigation.

### 4.3 Clinical implications

The results of our subgroup interaction analyses indicated that maternal age, BMI, and gestational week at testing did not exhibit significant interactions with the association between Lp(a) levels before 20 weeks and GDM. This finding robustly demonstrates that lower Lp(a) levels in early gestation are associated with an elevated risk of subsequent GDM, irrespective of maternal age or BMI.

In the subgroup sensitivity analysis, the association between serum Lp(a) levels at 12–20 weeks of gestation and GDM risk was attenuated. We postulate that this may be attributed to the physiological elevation of Lp(a) levels during the mid-to-late stages of pregnancy (35), which could diminish the intergroup differences between GDM and NGT subjects. Furthermore, no significant association was observed in subgroups with a BMI ≥23 kg/m² or maternal age >35 years. This phenomenon may occur because both elevated BMI and advanced age are well-established, independent risk factors for GDM (36, 37). Moreover, the effect of Lp(a) on GDM appeared to be weaker than that of BMI and maternal age, and its association was consequently obscured by these more dominant risk factors. Subgroup analyses may have been constrained by limited statistical power, particularly when sample sizes within subgroups were small, making it challenging to reliably detect true effect modification. Therefore, our subgroup analyses should be considered exploratory and cannot be interpreted as definitive evidence of effect modification. Future studies with larger sample sizes are warranted to validate these preliminary findings.

Our study did not assess the predictive utility of Lp(a) for GDM, as this would require formal prediction model analyses, which were beyond the scope of this etiologic investigation. whether adding Lp(a) to existing GDM prediction models improves their performance remains an open question for future research.

### 4.4 Strengths and limitations

It is widely acknowledged that randomized controlled trials (RCTs) provide high-level evidence, whereas retrospective studies face challenges in achieving randomized grouping comparable to that of RCTs. However, PSM can mitigate this issue by selecting and matching patients with similar baseline characteristics, thereby reducing confounding factors and approximating the conditions of an RCT (38). Although our study employed PSM on a large cohort to control for the effects of BMI, age, and Lp(a) measurement timing—thus enhancing the robustness of the evidence—certain limitations remain. We strictly adhered to the PSM protocol. However, after matching, a notable difference between groups in the gestational weeks of Lp(a) measurement persisted, which was unexpected. We attribute this finding to statistical significance driven by the large sample size rather than a genuine inter-group difference. Nonetheless, residual imbalance following PSM is possible, and we therefore additionally adjusted for this variable in the subsequent multivariate analysis.

In addition, our study is inherently limited by its design, and the findings may be subject to potential selection bias, residual confounding, and questionable adjustment choices that could affect the validity of our findings. This study lacks direct measures of insulin resistance, which precludes exploration of the underlying mechanisms linking Lp(a) to GDM, particularly the potential mediating role of insulin resistance. Future mechanistic studies should incorporate these measures.

A key strength of our study is the clear temporal sequence, with Lp(a) measured in early pregnancy (<20 weeks) prior to the clinical diagnosis of GDM, which typically occurs at 24–28 weeks. This temporal relationship supports the directionality of the observed association. However, we acknowledge that Lp(a) levels may fluctuate during pregnancy (35). While some studies suggest Lp(a) remains relatively stable in the first 20 weeks (39).

Notably, although 35% of the participants in this study were aged 35 years or older—a proportion that may appear elevated—this demographic characteristic aligns with the trend of delayed childbearing observed in the Chinese population. As the study was based on a general birth cohort, this age distribution does not constitute a study limitation.

## 5 Conclusion

In conclusion, low serum Lp(a) levels prior to 20 weeks of gestation are associated with higher odds of the development of GDM, regardless of pre-pregnancy BMI and maternal age. The risk is most pronounced when Lp(a) falls below 50 mg/L.

## Data Availability

The original contributions presented in the study are included in the article/supplementary material. Further inquiries can be directed to the corresponding authors.
